# Eldercare Responsibilities and Physical Health Symptoms Among Manufacturing Workers

**DOI:** 10.1093/geroni/igab046.1713

**Published:** 2021-12-17

**Authors:** Richard Fortinsky, Janet Barnes-Farrell, Jennifer Garza, Samantha Lacey, Hannah Austin, James Grady, Martin Cherniack

**Affiliations:** 1 University of Connecticut School of Medicine, Farmington, Connecticut, United States; 2 University of Connecticut, Storrs, Connecticut, United States

## Abstract

Working adults responsible for providing care to older relatives at home (eldercare) have reported greater psychological health problems such as depressive symptoms and stress than workers without eldercare responsibilities. Less is known about how eldercare is associated with physical health symptoms such as sleep-related problems and pain. Among manufacturing workers, such physical health symptoms have the capacity to diminish productivity. Therefore, we explored associations between eldercare responsibilities and physical health symptoms that could affect work performance in a sample of 357 adult employees from five manufacturing companies in a northeastern US state. Research questions were: are workers with eldercare responsibilities more likely than those without eldercare responsibilities to report sleep-related and pain-related symptoms, and are the number of eldercare tasks associated with these physical health symptoms? Among sample members, 52 (14.6%) provided eldercare, 62% were male, mean(standard deviation) age=49.8(12.7), and 77% were non-Hispanic White; no demographic differences were found between those with and without eldercare responsibilities. In bivariate analyses, we found that providing eldercare was associated with lower sleep quality (p=.05), fewer hours of sleep during the workweek (p=.04), more pain interference at home and at work (p=.02), and more pain on average in the past week (p=.01). Providing more types of eldercare tasks ranging from personal care to providing transportation was associated with more pain on average in the past week (p = .04). We conclude that eldercare is associated with physical health symptoms that could directly affect job performance among manufacturing workers. Workplace policy implications will be discussed.

